# Achieving SDG related sexual and reproductive health targets in China: what are appropriate indicators and how we interpret them?

**DOI:** 10.1186/s12978-020-00924-9

**Published:** 2020-06-01

**Authors:** Jing Fang, Shenglan Tang, Xiaoping Tan, Rachel Tolhurst

**Affiliations:** 1 Institute for Health Sciences, Kunming Medical University, 1168# Chun Rong Xi Lu, Yuhua Jiedao,Cheng Gong Qu, Kunming City, 50500 Yunnan Province PR China; 2 Duke Global Health Institute, 919-681-7760, 310 Trent Drive, Durham, NC 27710 Map Box 90519, Durham, NC 2770 USA; 3 Liverpool School of Tropical Medicine, Pembroke Place, Liverpool, L3 5QA UK

**Keywords:** Sexual and reproductive health, Sustainable development goals, Indicators, China

## Abstract

**Background:**

Sexual and Reproductive Health (SRH) targets have been included as part of the United Nations Sustainable Development Goals and indictors are important to monitor progress towards these targets. SRH indicators are recommended for setting norms and measuring progress globally. However, given the diverse political, socioeconomic and cultural contexts in different countries, and lack of global agreement on broad indicators, it is important to select appropriate indicators for specific countries. Based on internationally recommended indicators and data availability in China, this paper selected four indictors to reflect SRH in China and interpreted these indictors by analyzing the underlying factors.

**Methods:**

We employed secondary data analysis and key informant interviews. Secondary data were obtained from the China Health Statistical Yearbook (2005–2017), China Statistical Yearbook (2005–2017), and the sub-national estimates of the Global Burden Diseases Study 2016. We interviewed 36 key informants at national and sub-national levels.

**Results:**

The four selected SRH indicators are contraceptive prevalence rate (CPR), adolescent birth rate, abortion rate, and availability of school sex education. CPR of married women has remained above 75% over the last three decades, indicating a high level of access to family planning (FP) services; however, unmarried but sexually active women have significant unmet needs for FP services. Although adolescent birth rates in China remain low, the abortion rate, abortion numbers, and the ratio of abortions to births increased from 2014 to 2016 while FP policy was relaxed. This suggests that abortion among unmarried women is a significant contributor to overall figures. Qualitative analysis of the availability of school sex education, reveals an absence of policy due to conservative attitudes of key stakeholders.

**Conclusion:**

Since SRH challenges vary significantly between contexts, indicators for measuring progress towards SRH targets should be selected based on country context. The CPR and abortion rate are currently available and important indicators to monitor the most basic part of SRH in China, but require modification to ensure they reflect universal access to quality reproductive healthcare by all reproductive age women, regardless of their marriage status. Policy and indicators on sex education need to be carefully developed to fit the context in China.

## Plain English summary

Sexual and Reproductive Health (SRH) targets have been included as part of the United Nations Sustainable Development Goals and indictors are important to monitor the progress towards these targets. Based on internationally recommended indicators and data availability in China, we selected four indictors to reflect SRH in China.

We collected and analyzed data from the China  Health Statistical Year Book (2005–2017), China Statistical Year Book (2005–2017), and the sub-national estimates of the Global Burden Diseases Study 2016. We also interviewed 36 key informants at national and sub-national levels.

The four selected SRH indicators are contraceptive prevalence rate (CPR), adolescent birth rate, abortion rate, and availability of school sex education. We found that CPR of married women has remained above 75% over the last three decades, indicating women having good access to family planning (FP) services; however, this indicator does not cover unmarried but sexually active women. Meanwhile the abortion rate, abortion numbers, and the ratio of abortions to births increased from 2014 to 2016 while FP policy was relaxed in China. This suggests that abortion among unmarried women is a significant contributor to the overall figures. The lack of data on availability of school sex education, reveals a situation of absent policy and conservative attitudes of stakeholders. We recommend that In China, CPR and abortion rate need to be modified to cover unmarried people so as to reflect universal access to quality reproductive healthcare and indicators on sex education need to be carefully developed to fit the Chinese context.

## Introduction

In 2015, the United Nations Sustainable Development Goals (SDGs) succeeded the Millennium Development Goals (MDGs) as the new development agenda until 2030. Sexual and Reproductive Health (and Rights) were initially excluded from the MDGs and added following concerted advocacy by women’s health activists [1]. In contrast to the MDGs, which were critiqued for the reductionist approach to women’s health evident in the narrow scope of its goals and targets, SDGs have been welcomed as an opportunity to realize the expansive women’s health agenda emerging from the United Nations (UN) Conferences of the 1990s [2]. The 1994 International Conference on Population and Development (ICPD) Programme of Action adopted the first internationally recognized, normative definition of reproductive health, which incorporates aspects of physical, mental, and social well-being [3]. This states that “*reproductive health ... implies that people are able to have a satisfying and safe sex life and that they have the capability to reproduce and the freedom to decide if, when and how often to do so. Implicit in this last condition are the right of men and women to be informed and to have access to safe, effective, affordable and acceptable methods of family planning of their choice, as well as other methods of their choice for regulation of fertility which are not against the law... Reproductive health includes sexual health, the purpose of which is the enhancement of life and personal relations, and not merely counselling and care related to reproduction and sexually transmitted diseases*.” [4] Reproductive health (RH) is primarily incorporated in the SDGs under Goal 3 (target 3.7) and Goal 5 (target 5.6). Target 3.7 states: “By 2030, ensure universal access to sexual and reproductive health-care services, including for family planning, information and education, and the integration of reproductive health into national strategies and programmes”.

Despite valid critiques of metrics in global health [5], indicators remain a central driver of prioritization of effort and resources. It is therefore important that relevant and internally valid indicators are developed to enable all societal stakeholders, including state and civil society actors, to track progress, both nationally and internationally. As suggested by its history in the MDGs, RH has been described as “perhaps the most contentious target” in the SDGs [2], due to the significant polarization of social, political and religious values at play in its framing and interpretation. The key point of contention is whether women have a right to reproductive autonomy, and in particular whether they have a right to safe abortion. The contention undermines agreement on the appropriate normative content of SRH services. The resulting formulation of the target in rather broad terms has allowed for variation in its interpretation in practice and a lack of clear international agreement on meaningful indicators, between SRH advocates, technical and political actors, unlike other health areas such as non-communicable diseases and maternal and child health (MCH).

Efforts have been made at the international level to develop a comprehensive range of indicators for RH. One example is the set of Sexual and Reproductive Health and Rights (SRHR) Indicators for the SDGs recommended by the Guttmacher Institute [6], in which 18 indicators are recommended that correspond to three SDG goals: health (goal 3), education (goal 4) and gender equality (goal 5). These indicators cover nine topic areas: contraception, sexual and reproductive health service availability, knowledge about SHRH, adolescent fertility, quality of care (including respect for rights), prevention of STIs, abortion, comprehensive sexuality education and gender equality in SRHR. However, a much narrower set of indicators has been agreed at the international level to date. The list of indicators developed by the Inter-Agency and Expert Group on SDG Indicators (IAEG-SDGs) includes two for Goal 3.7: 1) 3.7.1 Proportion of women of reproductive age (aged 15–49 years) who have their need for family planning satisfied with modern methods; 2) 3.7.2 Adolescent birth rate (aged 10–14 years; aged 15–19 years) per 1000 women in that age group [7]. In addition, a closely related indicator is agreed for Goal 5: 5.6.2 Number of countries with laws and regulations that guarantee women aged 15–49 years access to sexual and reproductive health care, information and education.

There is undoubtedly merit in agreeing internationally comparable goals and targets for SRHR to develop normative benchmarks for principles and components, as well to monitor and compare progress among countries. However, there may be a range of barriers to the adoption of some indicators in some places in the short- or medium-term, which may include pragmatic limitations such as lack of data availability, as well as social, political or religious acceptability. Furthermore, since the challenges to achieving sexual and reproductive health may vary significantly between socio-economic, political and cultural contexts, efforts may be needed to identify the most appropriate indicators to guide and measure progress at national and sub-national levels. Therefore, whilst limiting from a normative perspective, the lack of universally agreed indicators for SRH arguably presents opportunities for countries to develop indicators that fit their own priorities. The aim of this paper is to identify and discuss indicators that are available and feasible in China, which can be used to drive direction and measure progress towards sexual and reproductive health (SRH) goals as defined in the SDGs.

In China, significant efforts have been made to reduce and ensure reliable measurement of the maternal mortality ratio as a key target for women’s health incorporated in the MDGs. Increasing access to institutional delivery has been accorded a high political priority, leading to well-documented, considerable achievements in reducing maternal mortality over the last 25 years [8], to the extent that the international targets set up by SDGs for reducing the maternal mortality ratio have already been met. Challenges remain to further reduce maternal mortality, especially in the poorer, rural and western areas of China and amongst vulnerable groups such as rural-to-urban migrants to meet the new MMR target set by the government. However, at the beginning of the SDG era it is timely to consider wider dimensions of reproductive health, particularly from a more holistic women’s health perspective. Sexual and reproductive health has received relatively little attention in policy or public health research in China in the last decade with the exception of family planning and HIV/AIDS prevention and control.

A family planning (FP) policy framework has been in place in China to restrict population growth since 1979, although this has undergone significant reforms over time. Two major recent changes are highly relevant to reproductive health. First, until 2013, FP policy was implemented by the well-funded Family Planning system (FP system), which controlled the authorization of births and provided free family planning and abortion services to married couples only. In 2013, the FP system was merged with the Ministry of Health to create a joint National Health and Family Planning Commission (renamed the National Health Commission in 2018). Second, a shift away from a highly controlled model of state-mandated long-term contraception towards an informed choice approach that allows couples to select contraceptives according to their needs culminated in 2016 in a significant policy change allowing all couples to have two children. This policy has been further relaxed in practice in some areas to allow some people to have a third child. Although this policy change was driven mainly by leadership concerns about the demographic reality of an aging population and shrinking labor force, the development of a quality of care program within the family planning service system since the year 2000 also responded to ICPD conference call [9]. These policy changes are likely to have significant effects on women’s reproductive health, both intended and unintended.

In this paper, we select four key indicators, namely contraceptive prevalence rate, adolescent birth rate, abortion rate, and school sex education**,** for measuring progress towards sexual and reproductive health (SRH) goals in China. Whilst there are a wide range of potential indicators available, we have focused on considering those developed by Inter-Agency and Expert Group on SDG Indicators (IAEG-SDGs) with regard to available data in China, as well as issues that emerged from key informant interviews and recent empirical literature as particularly relevant to consider in the Chinese context. We reflect on their meaning from a holistic women’s health perspective, in order to make recommendations for appropriate and meaningful indicators in the Chinese context in the short-to-medium term. To do so, we utilize both secondary quantitative data and primary qualitative data derived from key informant interviews with policy stakeholders.

## Methods

This paper was developed as part of a study aiming to develop evidence-based policy options for action to achieve the Health SDGs in China funded by The Bill and Gates Melinda Foundation (OPP: 1148464) [10]. In this paper, the major research methods employed were secondary data analysis and primary qualitative interviews.

Secondary data: We drew contraceptive prevalence rate of married women from the China  Health Statistical Year Book (2005–2017). Adolescent fertility rates were obtained from the sub-national estimates of the Global Burden Diseases Study 2016 conducted by Institute for Health Metrics and Evaluation (IHME). Total abortion numbers were drawn from the China Health Statistical Year Book (2005–2017) and we calculated the abortion rate by using the total number of reproductive age women (15–49) from China Statistical Year Book (2005–2017), as a denominator. Consistent with the over-arching study, we selected three provinces, namely Jiangsu, Hubei and Yunnan as representative of eastern, central and western region of China, in order to include diversity between provinces. The provinces were pragmatically selected according to access to informants through the networks of the research team members. We also collected and reviewed government RH-related policy and regulation documents including FP, MCH and school health education policies.

Primary data: We interviewed 36 key informants at national (8 persons, including 2 members of the National Commission of Health and Family Planning,^1^ 1 member of the Working Commission of Women and Children under the State Council, 1 retired senior official of formal International Cooperation Department of FP Commission, 1 senior researcher at the National FP Research Institute, 2 senior researchers from the National MCH Center and 1 official at the National Ministry of Education), provincial (11 persons; 3 in Jiangsu, 3 in Hubei and 5 in Yunnan), city and county level (17 persons in Yunnan). These key informants were selected according to their position, expertise and accessibility. We developed 16 interview guidelines for those interviewees with 6 for national level, 5 for provincial level and 5 for city and below level. The time spent for each interview varied from 40 min to 100 min with majority lasting for around 1 h. Informed consent was obtained from each interviewee beforehand and interviews were electrically recorded and transcribed into word documents and manually analyzed using a thematic analysis approach.

In addition, the paper is informed by the tacit knowledge of the first author, who has conducted substantial field research on various reproductive health issues across multiple contexts in China over a 25-year period, including participation in the quality of care program of FP services implemented by the former National FP commission. Her insights into the development of SRH policy and programming in China have informed the direction and contents of the paper.

A limitation of the methods is that we were not able to interview members of civil society, including representatives of NGOs working on SRHR or academics beyond the national MCH center and the National FP Research Institute, since the research was conducted as part of a wider study and resources were limited. However, we feel that our approach has two main strengths. First, interviewing informants from governmental and quasi- governmental (state research institutions) bodies at multiple levels provides ‘insider’ insights on the current situation and future prospects. Second, the triangulation of available quantitative data, including GBD data and national statistical data, with perceptions and explanations of trends provides a holistic picture of the issue.

## Results

In this section we present the data on the four key SRH indicators in China, interpret the data by incorporating findings from interviews, analyze the limits of current indictors caused by the changing socioeconomic and cultural context, particularly people’s attitude and behavior towards premarital sex, and make recommendations for future improvement.

### Contraceptive prevalence rate: what do the data say and how should we interpret them?

**Fig. 1:**
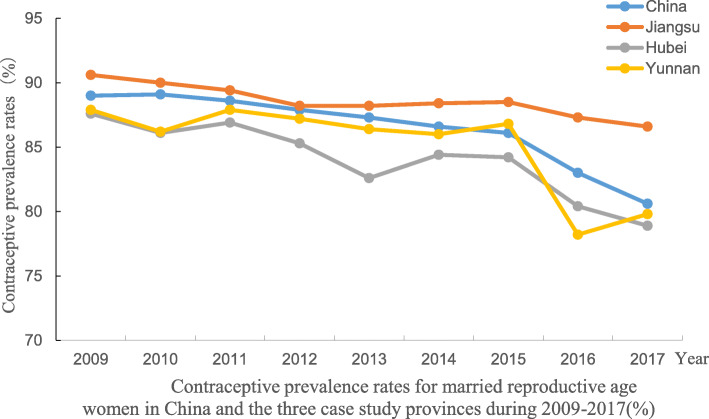
Contraceptive prevalence rates for married reproductive age women in China and the three case study provinces during 2009-2017(%)

One of SRH indicators suggested by the Inter-Agency and Expert Group on SDG Indicators for target 3.7 is 3.7.1 Proportion of women of reproductive age (aged 15–49 years) who have their need for family planning satisfied with modern methods. The framing of this indicator suggests the requirement for inclusion of information on ‘need for family planning’. In China, as in many contexts, data is available on the contraceptive prevalence rate, which is the total number of married women for whom use of modern contraceptives is reported, as a proportion of the total number of married reproductive age women assumed to be in need of family planning. Collection and reporting of this rate by the Family Planning Commission begun in the 1980s. The contraceptive prevalence rate has remained high over the past 3 decades. Figure 1 shows the high contraceptive prevalence rate (CPR) during the period 2010–2017 at national and the three sample provincial level. The rate has remained high to date, despite the impact of the merging of health and FP systems in 2013 and the changes to the “One child policy” in 2013 and 2016. Specifically, in 2013 the policy was relaxed to allow second births if one parent was an only child and then fully relaxed in 2016 to allow all couples to have a second child. This data clearly indicates a high level of access to FP services although it is heavily affected by the FP policy. However, a major limitation of the CPR according to data collected in China, and in many other countries, is that only married women are included. The complete absence of data on the CPR for unmarried, but sexually active women of reproductive age dates from a period when premarital sexual activity was socially unacceptable and relatively rare in a tightly controlled social context.

**Table 1:** Adolescent fertility rates in China and the three sample provinces during 1990–2016 (birth numbers per 1000 women)

Year	Age group	National	Jiangsu	Hubei	Yunnan
1990	10–14	0.08	0.05	0.08	0.14
	15–19	17.20	10.48	15.47	30.97
	Total	17.28	10.53	15.55	31.11
1995	10–14	0.06	0.04	0.05	0.09
	15–19	11.18	6.49	8.85	19.64
	Total	11.24	6.53	8.90	19.73
2000	10–14	0.04	0.03	0.04	0.07
	15–19	8.09	4.60	6.06	13.19
	Total	8.13	4.63	6.10	13.26
2005	10–14	0.04	0.03	0.04	0.05
	15–19	6.99	4.39	6.26	9.64
	Total	7.03	4.42	6.30	9.69
2010	10–14	0.03	0.03	0.04	0.04
	15–19	5.99	3.94	6.21	7.38
	Total	6.02	3.97	6.25	7.42
2016	10–14	0.03	0.02	0.03	0.03
	15–19	4.76	3.10	4.95	5.31
	Total	4.79	3.12	4.98	5.34

Data sources: the sub-national estimates of Global Burden of Diseases (GBD) Study 2016 conducted by IHME.

**Fig. 2:**
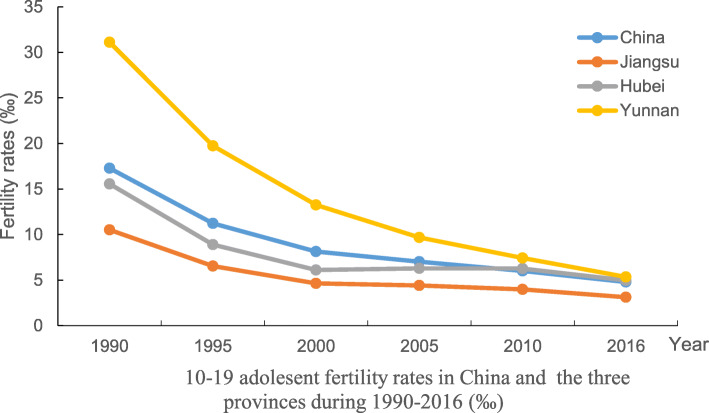
10-19 adolesent fertility rates in China and the three provinces during 1990-2016 (‰)

However, rapid economic and social change since the 1980s, including economic liberalization, migration, urbanization and globalization, has driven significant shifts in sexual attitudes and behavior [11–13]. There is now an increasing prevalence and acceptance of premarital sex and co-habitation without a marriage certificate in China, especially among young people and migrants. One national random sampling survey conducted in 2008 reported that 22.4% of unmarried 15–24 year old young people had sexual experience and 20.3% of them had more than one sexual partner in the last 12 months. Among the girls and young women with sexual experience, 21.3% had been pregnant, 4.9% more than once [14]. Other small scale surveys conducted in different regions of China revealed that premarital sex and cohabitation is common among young people [11–13]. It is clear that unmarried and sexually active young people, both women and men, have needs for contraceptives, although the full national picture of their access to contraceptives is unclear due to lack of data. However, there is some evidence of substantial unmet needs [15]. Universal access to FP services should cover unmarried but sexually active people. CPR is still a useful indicator in China to reflect people’s access to contraceptives, but its data collection should be revised to include unmarried but sexually active people. This requires reforms to the data collection and reporting system. The CPR of unmarried but sexually active people needs to be measured and monitored in order to fulfill the goal: universal access to FP services.

### Adolescent birth rate

Although the adolescent birth rate is not routinely reported in publicly available data, this information is available from the sub-national estimates of Global Burden of Diseases (GBD) Study 2016 conducted by IHME. GBD data shows that total adolescent fertility rates declined substantially between 1990 and 2016 in China as a whole, from 17.28 to 4.79, and in all three sample provinces (Table 1 and Fig. 2). Disparities between our sample provinces also declined, although the rate remained slightly higher in the poorest (Yunnan at 5.34) as compared to the richest province (Jiangsu at 3.12). At all time points, the vast majority of adolescent pregnancies occurred within the 15–19 year age group, which may be affected by the marriage law in China, which stipulates the minimum legal age of marriage for women as 18 years old in the first marriage law issued in 1950; this was raised to 20 years old in the revised version implemented since 1981. In addition, when FP policy was strictly implemented in China, it was hard for adolescent girls who were unmarried or did not have a marriage certificate to give birth to a child without punitive consequences. The 9 year compulsory education policy and programme, which provides free 9 years of education for both boys and girls, may also play an important role. The very low level of pregnancies amongst the 10–14 year old age group is welcome. However, since the legal age of marriage for women in China was 20 years for women throughout this period (although some women do get married and have children without a marriage certificate prior to the age of 20), the rate amongst 10–19 year olds is further indicative of unmet need for contraception among unmarried women. Although the data show a decline in the fertility rate to comparatively low levels internationally, the extent to which this reflects an improvement in the ability of young women to prevent unintended pregnancy is called into question by data on abortion rates, which we present in the next section.

### Abortion rates and their interpretation

In this paper, according to the available data, abortion rate is defined and calculated as the number of induced abortions per 1000 women between the ages 15 and 44 years. Safe abortion is a key dimension of sexual and reproductive rights. In many contexts therefore, attention and advocacy are appropriately focused on overcoming legal, social and health systems constraints to access by women to this service. However, with the exception of medical reasons, abortions are generally the result of unintended pregnancies. If people have good access to effective contraceptives so that the majority of pregnancies are planned and desired, and there are very few ‘hidden’ unsafe abortions, the abortion rate should be low. Therefore, in contexts such as China where safe induced abortion is legal and women have good access to safe abortion services, the abortion rate can be used as an indicator to reflect women’s access to and the quality of contraceptive services, since it implies that those women seeking abortions have either not used contraceptives or experienced contraceptive failure.

Table 2 shows the data on abortion rates in China between 2005 and 2016. It demonstrates an apparent increase in the abortion rate over the last 3 years to 28.13 per 1000 women in 2016. Compared with the estimated abortion rate of 35 per 1000 women in the world during 2010–2014, the abortion rate in China seems relatively low, close to the average of 27 in developed countries and lower than the average of 37 in developing countries and 34 in countries where abortion is legal [16]. However, key informant interview data reveals that the reported abortion numbers may be an underestimate. One key informant estimated that accurate numbers may be more than 10 million per year and one study reported that the annual abortion number in China is between 6 and 14 million [17]. If we use 6–14 million to calculate the abortion rate in China, it would be 17.49–40.83 and the upper limit would be higher than the international average level. One study attributes this gap between official and estimated figures to abortions carried out in private facilities [18]. According to this study, the officially reported abortion numbers were around 9 million in recent years while the numbers estimated by experts for the same time period were around 13 million, leading to a gap of 4 million. If we assume that the 4 million abortions were carried out in private facilities, then we could roughly estimate that 30% of all abortions occurred in private hospitals and clinics. Another major source of underreporting is that official data does not include the use of medical abortion (using mifepristone and misoprostol) when drugs are bought from pharmacies. There is no national level data on medical abortion, but two studies conducted in cities in Zhejiang and Fujian Provinces, reported that medical abortion accounted for 25 and 29% respectively of the total abortion conducted in the hospitals surveyed [19, 20]. Officially reported abortion data are not disaggregated by age, marital or socio-economic status, so it is not possible to identify which groups of women are particularly affected. Although it is not nationally representative, existing data from research projects suggests that abortion among young, unmarried women account for 30–60% of all abortions performed and one expert estimated that it accounts for 50% of the total abortion numbers [17].

**Table 2:** National abortion rate for reproductive age women (15–49) during 2005–2016 (‰)

Year	Abortion Numbers	Number of reproductive age women	Abortion rate(‰)
2005	7,105,995	351,623,083	20.21
2006	7,308,615	362,650,496	20.15
2007	7,632,539	360,900,000	21.15
2008	9,173,101	360,217,587	25.47
2009	6,111,375	363,526,919	16.81
2010	6,361,539	376,057,537	16.92
2011	6,631,310	382,937,647	17.32
2012	6,690,027	381,523,466	17.54
2013	6,237,177	375,750,608	16.6
2014	9,621,995	370,721,411	25.95
2015	9,851,961	365,669,032	26.94
2016	9,644,724	342,898,053	28.13

Data sources: the abortion rates in this table were calculated by dividing abortion numbers with the number of reproductive age women of the same year. Abortion numbers come from “China Health Statistical Yearbook 2006-2012” and “China Health and Family Planning Statistical Yearbook 2013-2017”, and number of reproductive age women come from “China Statistical Yearbook 2008-2017”.

As discussed above, there are limitations on the reliability of the existing data on abortion rates. However, it may be useful to consider the scale of reported abortion numbers in the context of births, by comparing the ratio of abortion numbers to numbers of live births. The birth data is more reliable due to the improved vital registration system and almost 100% institutional delivery in China. The ratio of abortion numbers to birth numbers at the national level also increased during 2014–2016 and in Jiangsu province in most of years between 2005 and 2016 this ratio is greater than 1, meaning that abortions outnumber births (Table 3). It is worth noting that due to the 2013 and 2016 relaxation of the FP policy allowing couples to have two children, it might be expected that abortion numbers would decrease, but in fact abortion numbers in the whole country and the three sample provinces, namely, Jiangsu, Hubei and Yunnan Province increased. This may suggest that abortions among unmarried women are a significant contributor to the overall figures, since the change in FP policy has little impact on this population group. Many research studies conducted in different part of China showed that repeated abortions account for more than half of the total number of abortions [21–23].

**Table 3:** the ratio of abortion number to birth number in the whole country and three provinces during 2005–2016

	Nation	Jiangsu	Hubei	Yunnan
2005	0.62	1.36	0.59	0.48
2006	0.62	1.35	0.57	0.49
2007	0.61	1.25	0.57	0.53
2008	0.69	1.22	0.59	0.72
2009	0.44	0.68	0.41	0.38
2010	0.45	0.75	0.38	0.36
2011	0.46	0.74	0.39	0.41
2012	0.43	0.72	0.35	0.37
2013	0.41	0.74	0.35	0.36
2014	0.63	1.14	0.45	0.69
2015	0.68	1.10	0.53	0.83
2016	0.52	0.88	0.51	0.65

Data sources: the ratio of abortion number to birth number was calculated by dividing the abortion number with birth number of the same year. Both the numbers of abortion and birth come from “China Health Statistical Yearbook 2006-2012” and “China Health and Family Planning Statistical Yearbook 2013-2017”.

It is important to consider the implications of these numbers carefully. Although it is a reproductive right, abortion is a medical intervention that carries potential risks for women’s health and their future fertility, particularly in the case of repeat abortions. From a women’s health perspective therefore, the use of abortion as a contraceptive should be avoided where possible, through meeting women’s needs for accessible and effective contraceptive methods. Furthermore, it is concerning if abortion provision is at the expense of contraceptive services due to commercial or financial considerations. The health financing system in China has introduced a perverse incentive for the provision of abortion services over providing good quality contraceptive services. Funding from government budget are less than 10% of the total expenditure in most public hospitals requiring them to generate operating costs through fees for services [24]. As a medical service, abortion, particularly abortion with anesthesia, is therefore a significantly more profitable service than family planning services. For example, in some MCH hospitals, income from the FP service department, which mainly provides abortion services, can contribute around half of the hospital total service income. Key informants thought that the promotion of ‘painless abortion’ through commercial advertising presents abortion as a ‘risk-free’ procedure, and this is particularly influential among young women. While ensuring entitlements and access to legal, safe abortion, reproductive health policy should prioritize promoting women’s health through contraceptive provision as a social good, rather than allowing a market logic to dominate at the expense of women’s health.

### Sex education

Comprehensive sex education is an important component of RH and is also an explicit SDG target under the education goal (target 4.7). There is strong international evidence that sex education can reduce unwanted pregnancy and associated abortion and prepare young people to better prevent STIs [25]. This indicator is therefore strongly linked to the above indicators of contraceptive prevalence, adolescent fertility and abortion. However, it is still contentious in many countries in terms of what the contents should be and who should provide sex education to whom via what channels and when. In China, sex education can be considered as the weakest link among all RH services despite the fact that many young people obtain sex information from multiple sources, including the internet and their peers. Although it is mentioned in several laws and policies, there is no specific policy on sex education and comprehensive sex education is rarely provided in schools. Given the importance of sex education in RH, it is crucial to develop indicators in China to measure and monitor the progress towards SDG goals.

The indicator recommended by the Guttmacher Institute is “proportion of schools that serve students in the age range of 12-17 years in which comprehensive sexuality education is available.” If this indicator was used in China, the first step would be the defining the contents of comprehensive sexuality education. Ideally, the more comprehensive the contents the better; however currently many schools, particularly those located in less developed provinces, face shortages of teachers and teaching materials including text books, and limited teaching time for sex education given the already very crowded school curriculums. More importantly, the schools run the risk of a societal backlash for teaching children knowledge that is considered inappropriate for them by society at large. Thus, the first and currently more important indictor on sex education for China should be whether a specific policy on school sex education is available. This school sex education policy should cover the questions raised in the beginning of this section and define the contents of comprehensive sex education based on the Chinese context. Once this policy is in place, it will be more meaningful to measure how many schools in China offer comprehensive sexuality education to students.

Interview data shows that all stakeholders including health care providers, health department officials, education department officials and researchers are aware of the issues of premarital sex and unwanted pregnancies and associated abortion among young people and also acknowledge the importance of sex education. However, few stakeholders are currently willing to initiate policy development or provide school sex education beyond abstinence advice, including the education department, which takes a very conservative approach. There are several reasons for this, but principally officials fear being held responsible for any unwanted consequences of sex education. For example, one county level education department official said:

*“It is better not to touch [sex education]. If you do it, you may make more students explore sex.”*

One national level officer reported:

*“We sent an expert team to other countries to learn about school sex education, but we are still not sure about the extent of sex education for school children.”*

The Health Education Guideline for the General University and College issued by the Ministry of Education in 2017 prescribed relatively comprehensive sex education for university students but there is little content on sex education in the Health Education Guideline for Middle and Primary school. One Key informant from the Ministry of Education emphasized the shared responsibility for sex education between a range of societal stakeholders by saying:

*“Sex education should not be a task only for education department and schools; parents, mass media and the health sector should share the responsibility”.*

A specific school sex education policy is an essential first step as a signal of official approval, at this moment when sex education is very limited and all stakeholders are hesitating. However, this indicator is definitely insufficient to guarantee the availability of comprehensive, non-judgmental, gender equitable sex education for all those who need it, regardless of sexual orientation or gender identity.

## Discussion: implications for action

The inclusion of SRH in the SDG goals represents great progress in the human development agenda. In comparison to many other health issues, SRH is very broad, covering many different but interconnected issues, and is also contentious. Thus, indicators are crucial for realizing SRH goals since they can be used to pinpoint the direction where efforts are needed and to monitor the progress toward that direction. However, despite the internationally agreed definition, SRH in different socioeconomic and cultural contexts has been understood and interpreted differently and is subject to contention, particularly with regard to sexual and reproductive rights. In addition, the urgent issues and the priorities in SRH may be different in different countries. It is therefore pragmatic and imperative to develop indicators that are appropriate to both drive agendas and measure progress in specific countries.

In China, based on the existing data collection and reporting system, we recommend reforming the three indicators discussed above, namely contraceptive prevalence rate, abortion rate, and school sex education policy, as a starting point for developing a more comprehensive set of SRH indicators in future. The current data collection for the contraceptive prevalence rate and abortion rate should be expanded to cover unmarried reproductive age population and the calculation of the two indicators should be disaggregated by marriage status, age, migrant status, socioeconomic status and other meaningful variables. The health information system should cover the private hospitals and clinics so that abortions conducted by these medical institutions can be included. Further, the contraceptive prevalence rate has been critiqued as an insufficiently sensitive to client-oriented service dimensions [26]. Historically a range of barriers to effective implementation of client-centered contraceptive services have been identified in China [27–29]; however, there is a dearth of recent studies. It will therefore be necessary to develop meaningful indicators of service quality from user perspectives in the future.

China is a signatory to the Global Strategy for Women, Children, and Adolescent Health and has committed itself under this global framework to tackle adolescent reproductive health. This provides a mandate for further development of school sex education, its use as an indicator and expansion of its content. Society-wide action, including civil society and across multiple sectors, may be required to create the conditions for this. Furthermore, policies that contribute towards difficulties for women who have a child outside of marriage by restricting entitlements and benefits for their children may contribute towards abortions for young women in particular. They should therefore be re-considered as part of a strategy to promote adolescent reproductive health.

The combined analysis of the three indicators can be used to identify unmet needs for contraception in different population groups and to roughly assess the effect and quality of FP services. As reflected by the current status of the two existing indicators, quality FP services should be maintained and strengthened to provide contraceptives for married and unmarried women and their male partners so as to reduce unintended pregnancies and unnecessary abortions, and thus contribute to improve women’s health. It is especially important to strengthen FP services in current situation, because the population control policy has been relaxed, so FP services may not receive the previous level of support from the government. In addition, there is a crucial need to address the perverse financial incentive for health services to provide abortion services over contraceptive services by strengthening the mandate and funding for contraceptive services as part of preventive health services in primary health care.

## Conclusion

In conclusion, in the absence of agreement on a wide set of SRH indicators to measure the SDGs, it is important to ensure that agreed indicators are appropriate and measurable in specific country contexts. In China, the contraceptive prevalence rate and abortion rate are currently available and important indicators to monitor the most basic part of SRH, but require modification to ensure they are fit for the purpose of ensuring universal access to quality reproductive healthcare by all reproductive age women regardless their marriage status. Furthermore, the perverse financial incentives for health facilities to maintain high abortion rates, which place adolescent women at particularly high risk, require urgent policy attention. More detailed indicators on sex education that can cover comprehensive contents are also desperately needed in China to monitor progress towards SDG SRH targets and they need to be carefully developed to fit the context in China.

## Data Availability

Contraceptive prevalence rate, abortion numbers, birth numbers, and numbers of reproductive age women used in this paper were obtained from openly published China Health Statistical Year Book (2005–2017) and China Statistical Year Book (2005–2017). Adolescent fertility rates were obtained on request from the sub-national estimates of the Global Burden Diseases Study 2016 conducted by Institute for Health Metrics and Evaluation (IHME).

## Abbreviations

CPR: Contraceptive prevalence rate
FP: Family planning
MCH: Maternal and Child Health
MDGs: Millennium Development Goals
SDGs: Sustainable Development Goals
RH: Reproductive Health
SRH: Sexual and Reproductive Health
SRHR: Sexual and Reproductive Health and Right

## Footnotes

1 It was renamed in 2018 as The Health Commission.
